# Reparative Effects of a Topical Antioxidant Serum Containing Vitamin C, Vitamin E, and Ferulic Acid After Ablative Fractional CO_2_ Laser Treatment for Atrophic Acne Scars: A Randomized, Investigator‐Blinded, Split‐Face, Controlled Trial

**DOI:** 10.1111/jocd.70634

**Published:** 2026-01-11

**Authors:** Yu Shi, Sijia Xu, Wei Zhang

**Affiliations:** ^1^ Department of Medical Cosmetology, Shanghai Skin Disease Hospital, School of Medicine Tongji University Shanghai China

**Keywords:** ablative fractional CO_2_ laser, aesthetic dermatology, atrophic acne scars, CE Ferulic serum, cosmetic dermatology, evidence‐based medicine, integrated skincare

## Abstract

**Background:**

Ablative fractional CO_2_ laser is effective for acne scar treatment but is often associated with side effects such as erythema and dyspigmentation, along with prolonged recovery time.

**Aims:**

The study aimed to evaluate the post‐procedure reparative effects of a combination of vitamin C, E, and ferulic acid (CE Ferulic) in Chinese patients with atrophic acne scars.

**Methods:**

In this randomized, investigator‐blinded, split‐face, controlled trial, patients aged 18–50 with moderate‐to‐severe atrophic acne scars were randomly assigned to apply CE Ferulic to intervention‐side face and normal saline (NS) to control‐side face, immediately after ablative CO_2_ laser treatment for 14 days. Patients were further divided into once‐daily and twice‐daily application subgroups. During the 14‐day follow‐up, the wound healing (measured by scabbing stage on Day 7), erythema index (EI), melanin index (MI), skin hydration, and transepidermal water loss (TEWL) were evaluated. Direct assessment of scar improvement was not within the scope of this study.

**Results:**

Sixty‐four patients were included in the analysis. On Day 7, the intervention side showed a higher complete scab detachment rate than the control side (60.9% vs. 34.4%, *p* = 0.0026). EI and MI reduced significantly greater on the intervention side compared to the control side (Days 3, 7, and 14; *p* < 0.0001). On Day 14, the intervention side also demonstrated significantly better capability of maintaining skin hydration (*p* = 0.0367) and preventing TEWL (*p* = 0.0246) than the control side.

**Conclusions:**

This study found that CE Ferulic led to enhanced wound healing, reduced erythema and melanin levels, and improved skin hydration following laser treatment, suggesting its beneficial application in combination with laser treatment to accelerate skin recovery.

**Trial Registration:**

ChiCTR2300078214

## 1 Introduction

Acne scarring is a prevalent sequelae resulting from aberrant collagen production during the natural healing process of inflammatory acne lesions, with atrophic acne scars being the most common type [1]. Ablative fractional carbon dioxide (CO_2_) lasers are a widely used treatment that achieves significant improvement of facial atrophic acne scars through removing damaged scar tissues on the skin surface as well as tightening the collagen fibers beneath [2]. However, this procedure is associated with numerous side effects including erythema, dyspigmentation, telangiectasia, and lengthy recovery time, and can have negative impacts on patients' quality of life [3]. Evidence has shown that Asian skin is more prone to post‐inflammatory hyperpigmentation and scar formation than Caucasians [4, 5, 6]. As a result, there remain unmet needs for minimizing side effects and shortening recovery time following ablative CO_2_ laser treatment, especially in Asian populations.

In recent years, the concept of Integrated SkinCare has been proposed to promote combining clinical aesthetic procedures with dermatological skincare products and advanced home care, which aims to deliver long‐lasting and comprehensive results of skincare [7, 8, 9]. Antioxidants have been shown to be effective in mitigating the side effects of laser treatment and improving overall outcomes. Vitamin C is a potent antioxidant, counteracting and eliminating oxidative stress that often occurs following ablative lasers. It also promotes collagen synthesis and plays a vital role in the wound healing process [10]. The combination of vitamin C with vitamin E has also been proven to reduce oxidative damage to the skin compared to the vehicle solution [10, 11, 12].

Previous clinical trials have demonstrated that a combination of vitamin C, vitamin E, and ferulic acid (which can stabilize the formulation of vitamin C and E) [13] is associated with decreased postoperative erythema and edema, reduced skin pigmentation, and improved wound healing compared with vehicle in patients who have undergone laser treatment [14, 15]. A chart review study also illustrated a favorable safety profile of the application of the combination of vitamin C, vitamin E, and ferulic acid following ablative fractional laser treatment [16]. However, evidence regarding the post‐laser recovery effect of this formulation is scarce within the Chinese population. To address this gap, this trial aims to assess the postoperative reparative effects of an antioxidant serum, known as CE Ferulic serum, after ablative fractional CO_2_ laser treatment in the Chinese population with atrophic acne scars compared with a placebo comparator (normal saline).

## 2 Materials and Methods

### 2.1 Study Design and Patients

This study is a randomized, investigator‐blinded, split‐face, controlled trial. The enrollment period lasted for 60 days from December 2020 to January 2021.

Patients aged 18–50 years old with moderate‐to‐severe atrophic acne scars on their face were eligible to participate. The severity of atrophic acne scars was assessed using échelle d'évaluation clinique des cicatrices d'acné (ECCA) grading scale [17], with a score of 16–45 being moderate and a score of 46–80 being severe. Individuals were excluded if (1) they were make‐up users (excluding lip gloss, lipstick, eyeliner, or eyeshadow); (2) they were allergic to any component in the trial serum. Written informed consent was obtained from each subject.

### 2.2 Interventions

All patients received symmetrical treatment using ablative fractional CO_2_ laser (CO2RE; Candela Inc.) on both sides of their face, with identical depth (DEEP mode), energy (55 J/cm^2^), pattern size (6.7 × 6.7 mm), fractional coverage (5%), and repeat interval (0.25 s). Immediately after the laser treatment, each subject was instructed to apply the serum on one side of their face, which contained vitamin C, vitamin E, and ferulic acid (CE Ferulic; SkinCeuticals Inc., #74001921), while the other side was treated with placebo—0.9% sodium chloride (normal saline, NS). Patients applied the serum/NS once daily in the morning or twice daily (morning and night) for 14 days determined by practitioners.

### 2.3 Randomization

The assignment of sides of the face was randomized before conducting the experiment. A random number generator in Microsoft Excel (2019 version; Microsoft) was used to generate a series of 0s or 1s. Each random assignment was then sealed in a separate nontransparent envelope. The assignments were made in consecutive order to determine which side of the face would receive the CE Ferulic serum.

### 2.4 Endpoints and Follow‐Up

Patients were observed and evaluated at baseline (prior to procedure), post‐procedure (30 min after serum application immediately following procedure), Days 3, 7, and 14. Investigators were blinded to the treatment conditions to ensure unbiased evaluation when they conducted the measurements and assessments of the skin parameters.

The primary endpoints of this study were wound healing and erythema index (EI). Wound healing was assessed by the percentages of patients under each stage of scabbing. On Day 7, patients were grouped into one of three scab stages by blinded investigators: scab formation, partial scab detachment, and complete scab detachment [18]. The EI was evaluated using a Mexameter MX 18 connected to a Multiprobe Adapter System 9 (MPA 9) (Courage+Khazaka electronic GmbH).

The secondary endpoints included the melanin index (MI), skin hydration, transepidermal water loss (TEWL), and skin sebum content. The MI was measured using a Mexameter MX 18 as stated above. Skin hydration was assessed using a Corneometer CM 815. TEWL (g/m^2^/h) was measured using a Tewameter TM 300. Skin sebum content was measured using a Sebumeter SM 815. Clinical photographs of the skin were obtained using the VISIACR Photo Station (Canfield Imaging Systems, Fairfield, NJ, USA).

### 2.5 Statistical Analysis

The required sample size in the final analysis was 46, given a two‐tailed test with a significance level of 0.05, a desired power of 0.9, a standard deviation of 1, and an acceptable error of ±1. To account for potential dropouts, a total of 66 patients were recruited, with a ratio of 1:1 in each subgroup (once‐daily and twice‐daily).

For continuous variables including the EI, MI, skin hydration, skin sebum content, and TEWL, paired *t*‐tests were used for split‐face comparisons between the treatment side and the control side. For the categorical variable (i.e., wound healing), chi‐square tests were performed. Subgroup analysis was conducted to explore the efficacy and tolerability of CE Ferulic serum in each subgroup (once‐daily and twice‐daily subgroups).

All statistical analyses were performed using SPSS version 23.0 (SPSS Inc). A significance level alpha < 0.05 was considered statistically significant in all cases.

## 3 Results

**FIGURE 1:**
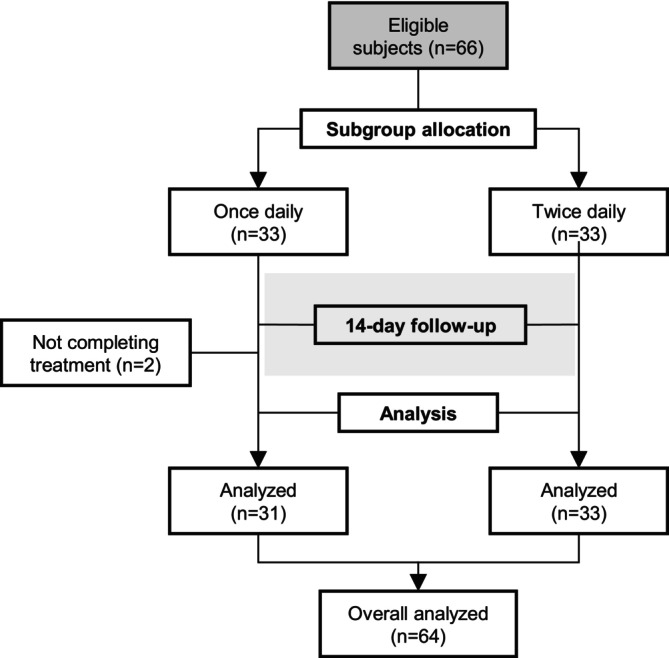
Flowchart of study design.

Out of 66 patients included in the study, 33 were assigned to the once‐daily subgroup, and the remaining 33 were assigned to the twice‐daily subgroup. Two patients in the once‐daily subgroup failed to complete the entire course of treatments and thus were not included in the analysis (Figure 1). Among the 64 patients included in the analysis, 44 (68.8%) were female. The mean (SD) age of the patients was 29.5 (5.31) years. All patients had Fitzpatrick skin type IV.

### 3.1 Wound Healing—Stage of Scabbing

All patients started to develop scabs by Day 3 and were observed to be fully detached by their last visit on Day 14. On Day 7, for the side applying CE Ferulic serum, 39 (60.9%) patients achieved complete scab detachment, significantly higher than the NS side of 22 (34.4%) (*p* = 0.0026). In subgroup analysis, the trend remained regardless of the frequency of CE Ferulic serum/NS application. The CE Ferulic‐treated side achieved a significantly higher proportion of complete scab detachment compared with the NS side in the once‐daily subgroup (61.3% vs. 29.0%, *p* = 0.0107), while the effect was not statistically significant in the twice‐daily subgroup (*p* = 0.0848) (Table 1). Stage of scabbing on Days 3, 7, and 14 overall and by subgroup were recorded in Table S1.

**TABLE 1:** Stage of scabbing on Day 7.

Stage of scabbing	Overall			Once‐daily subgroup			Twice‐daily subgroup		
	Serum	NS	*p*	Serum	NS	*p*	Serum	NS	*p*
Scab formation, *n* (%)	0 (0%)	1 (1.6%)	0.0026*	0 (0%)	1 (3.2%)	0.0107*	0 (0%)	0 (0%)	0.0848
Partial scab detachment, *n* (%)	25 (39.0%)	41 (64.1%)		12 (38.7%)	21 (67.7%)		13 (39.4%)	20 (60.6%)	
Complete scab detachment, *n* (%)	39 (60.9%)	22 (34.4%)		19 (61.3%)	9 (29.0%)		20 (60.6%)	13 (39.4%)	

*Note:* *p* values were derived from the chi‐square test comparing subject distribution in “Partial scab detachment” and “Complete scab detachment” between the two face sides.

*p < 0.05.

### 3.2 Erythema Index

**FIGURE 2:**
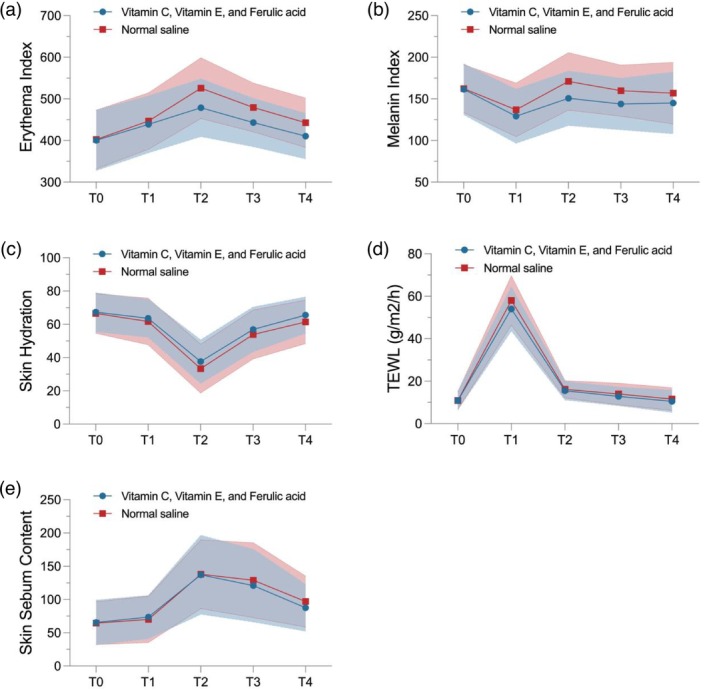
Skin parameters at baseline and during follow‐up. (a) Erythema index; (b) melanin index; (c) skin hydration; (d) TEWL; (e) skin sebum content. T0, baseline; T1, post‐procedure; T2, day 3; T3, day 7; T4, day 14; TEWL, transepidermal water loss. The blue‐ and red‐shaded areas represent the ranges between the standard deviation added and subtracted from the mean value in CE Ferulic and normal saline group, respectively.

Both face sides exhibited an upward trend in EI from baseline and peaked on Day 3, followed by a subsequent decrease, suggesting the erythema level experienced an increase on the first few days after laser treatment, and damped down afterwards. The CE Ferulic‐treated side had lower EI than the NS side throughout the follow‐up period (Figure 2a). The CE Ferulic‐treated side experienced a significantly lower increase from baseline in EI compared to the NS side on Days 3, 7, and 14, regardless of the frequency of serum application. The differences (SD) in the changes from baseline between the CE Ferulic side and the NS side were −11.1% ± 9.18% (*p* < 0.0001), −8.6% ± 8.22% (*p* < 0.0001), and −7.6% ± 5.69% (*p* < 0.0001) on Days 3, 7, and 14, respectively (Table 2). There was no significant difference in EI observed between the CE Ferulic side in the two subgroups throughout the follow‐up.

**TABLE 2:** Mean percentage changes from baseline in skin parameters.

Skin parameters	Overall (*N* = 64)				Once‐daily subgroup (*N* = 31)				Twice‐daily subgroup (*N* = 33)			
	Mean changes from baseline (%)		Difference between groups, mean ± SD (%)	*p*	Mean changes from baseline (%)		Difference between groups, mean ± SD (%)	*p*	Mean changes from baseline (%)		Difference between groups, mean ± SD (%)	*p*
	CE‐F	NS			CE‐F	NS			CE‐F	NS		
**Erythema index**												
T1: post‐procedure	10.5	12.0	−1.5 ± 6.02	0.0553	11.0	13.0	−2.0 ± 6.48	0.0956	10.1	11.1	−1.0 ± 5.90	0.2955
T2: Day 3	21.0	32.1	−11.1 ± 9.18	< 0.0001***	24.2	36.3	12.11 ± 8.92	< 0.0001***	18.0	28.3	−10.3 ± 9.48	< 0.0001***
T3: Day 7	12.6	21.2	−8.6 ± 8.22	< 0.0001***	16.7	25.4	−8.7 ± 8.42	< 0.0001***	8.8	17.3	−8.5 ± 8.19	< 0.0001***
T4: Day 14	4.3	11.9	−7.6 ± 5.69	< 0.0001***	6.2	13.5	−7.3 ± 5.44	< 0.0001***	2.5	10.4	−8.1 ± 5.98	< 0.0001***
**Melanin index**												
T1: post‐procedure	−19.8	−15.6	−4.2 ± 10.92	0.0027**	−20.2	−15.9	−4.3 ± 10.01	0.0219	−19.5	−15.3	−4.2 ± 11.86	0.0507
T2: Day 3	−5.8	5.9	−11.7 ± 13.86	< 0.0001***	−6.7	6.5	−13.2 ± 14.85	< 0.0001***	−5.0	5.3	−10.3 ± 12.92	< 0.0001***
T3: Day 7	−10.1	−0.8	−9.3 ± 13.32	< 0.0001***	−12.5	−0.5	−12.0 ± 15.07	0.0001**	−7.9	−1.1	−6.8 ± 11.08	0.0013**
T4: Day 14	−10.3	−3.6	−6.7 ± 11.98	< 0.0001***	−8.6	−2.9	−5.7 ± 9.63	0.0025**	−11.8	−4.3	−7.5 ± 13.93	0.0042**
**Skin hydration level**												
T1: post‐procedure	−2.6	−4.9	2.3 ± 20.41	0.3586	−2.8	−4.0	1.2 ± 18.9	0.7249	−2.3	−5.8	3.5 ± 21.98	0.3748
T2: Day 3	−43.2	−48.5	5.3 ± 17.09	0.0161*	−44.6	−48.5	3.9 ± 18.15	0.2476	−41.9	−48.5	6.6 ± 16.19	0.0248*
T3: Day 7	−13.1	−17.0	3.9 ± 16.15	0.0585	−17.4	−19.4	2.0 ± 16.92	0.5055	−9.0	−14.6	5.6 ± 15.45	0.0446*
T4: Day 14	0.2	−4.8	5.0 ± 18.48	0.0367*	−4.1	−6.8	2.7 ± 19.91	0.4614	4.2	−2.9	7.1 ± 17.07	0.0236*
**TEWL**												
T1: post‐procedure	470.7	503.6	−32.9 ± 134.17	0.0543	460.7	511.7	−51.0 ± 165.91	0.0976	480.0	495.9	−15.9 ± 95.00	0.3428
T2: Day 3	66.2	70.4	−4.2 ± 42.95	0.4445	53.9	63.1	−9.2 ± 42.84	0.2426	77.8	77.2	0.6 ± 43.17	0.9364
T3: Day 7	39.2	45.7	−6.5 ± 41.60	0.2186	33.2	42.4	−9.2 ± 43.25	0.2451	44.9	48.8	−3.9 ± 40.49	0.5857
T4: Day 14	6.1	15.0	−8.9 ± 31.05	0.0246*	1.6	10.4	−8.8 ± 26.47	0.0751	10.3	19.4	−9.1 ± 35.23	0.1477
**Skin sebum content**												
T1: post‐procedure	46.8	41.6	5.2 ± 36.25	0.2604	74.8	64.7	10.1 ± 38.67	0.1587	20.4	19.9	0.5 ± 33.77	0.9261
T2: Day 3	160.0	161.3	−1.3 ± 81.70	0.9044	185.4	187.9	−2.5 ± 95.11	0.8864	136.2	136.3	−0.1 ± 68.24	0.9949
T3: Day 7	122.7	134.2	−11.5 ± 60.36	0.1342	145.0	151.8	−6.8 ± 55.44	0.4997	101.8	117.6	−15.8 ± 65.19	0.1732
T4: Day 14	60.6	81.2	−20.6 ± 48.78	0.0012**	80.0	104.6	−24.6 ± 37.76	0.0011**	42.3	59.2	−16.9 ± 57.61	0.1016

*Note:* *p* values were derived from paired *t*‐test comparing difference between groups and zero.

Abbreviations: CE‐F, CE ferulic serum; NS, normal saline; SD, standard deviation; TEWL, transepidermal water loss.

*p < 0.05; **p < 0.01; ***p < 0.001.

### 3.3 Melanin Index

The CE Ferulic serum side consistently exhibited a lower MI than the NS side throughout the study period (Figure 2b). From post‐procedure to Day 14, the serum side demonstrated a significantly greater reduction from baseline in MI compared to the NS group overall, and in both the once‐daily and twice‐daily subgroups. The differences in the changes from baseline in MI between CE Ferulic side and NS side were −4.2% ± 10.92% (*p* < 0.0027), −11.7% ± 13.86% (*p* < 0.0001), −9.3% ± 13.32% (*p* < 0.0001), and −6.7% ± 11.98% (*p* < 0.0001) at post‐procedure, Days 3, 7, and 14, respectively (Table 2). No significant difference in MI between CE Ferulic‐treated side in two subgroups was found.

### 3.4 Skin Hydration

The level of skin hydration for both treatment groups dropped instantly following the laser treatment until Day 3, after which it gradually increased until Day 14 (Figure 2c). The CE Ferulic serum group exhibited a superior capability in maintaining skin hydration compared to NS on Day 3 (5.3% ± 17.09%, *p* = 0.0161) and Day 14 (5.0% ± 18.48%, *p* = 0.0367). Specifically, when applied twice daily, the skin hydration level increased by 4.2% from baseline in the CE Ferulic group, while the NS group experienced a 2.9% decrease on Day 14 (*p* = 0.0236). There was no significant difference in the percentage change in skin hydration from baseline between the CE Ferulic serum and the NS sides in the once‐daily subgroup (Table 2).

### 3.5 Transepidermal Water Loss (TEWL)

TEWL reached its peak at the time of post‐procedure (Figure 2d). On Day 14, the CE Ferulic‐treated side showed a significantly lower percentage increase from baseline in TEWL compared with the NS side (−8.9% ± 31.05%, *p* = 0.0246). There was no significant change in TEWL from baseline to any follow‐up time point in either once‐daily or twice‐daily subgroup (Table 2).

### 3.6 Skin Sebum Content

The skin sebum content initially showed an upward trend after laser treatment, reaching its peak on Day 3, and then gradually decreased thereafter. The levels of skin sebum on both the CE Ferulic side and the NS side were similar until Day 3, but began to diverge thereafter (Figure 2e). The serum group exhibited a significantly lower percentage increase from baseline compared to the NS group on Day 14 (−20.6% ± 48.78%, *p* = 0.0012). In subgroup analysis, the result remained significant in the once‐daily subgroup but not significant in the twice‐daily group (Table 2).

### 3.7 Safety and Tolerability

During the 2‐week study period, no adverse drug reactions were reported. The application of the CE Ferulic serum following ablative CO_2_ laser treatment showed an overall great tolerability in all patients.

**FIGURE 3:**
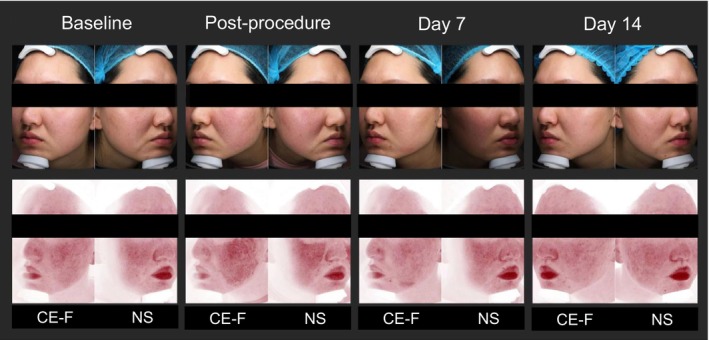
Clinical photographs of the skin on both sides of the face at baseline and during follow‐up. CE‐F, CE Ferulic serum; NS, normal saline.

Clinical photographs of both sides of the facial skin at baseline, post‐procedure, on Days 7 and 14 are also shown (Figure 3).

## 4 Discussion

This study is the first randomized, investigator‐blinded, split‐face, placebo‐controlled trial in China to assess the postoperative reparative effects of a combination of vitamin C, vitamin E, and ferulic acid as an adjunct to ablative CO_2_ laser treatment among patients with atrophic acne scars.

The photothermal effect of the laser damages the barrier function of the skin while being treated, leading to a variety of complications such as erythema, dyspigmentation, skin dehydration, and sometimes even oozing from the wound surface [19]. The current study found that an optimal combination of antioxidants resulted in rapid healing from the damage following ablative laser treatment, compared with NS. The serum also had a greater erythema reduction ability throughout the first 2 weeks following laser treatment, regardless of whether the treatments were applied once or twice daily. In a randomized, placebo‐controlled study conducted in the US, a combination of vitamin C, vitamin E, and ferulic acid demonstrated faster wound healing represented by reduced erythema and edema in the following 7 days after ablative laser treatment, in agreement with our trial [14]. Furthermore, a recent study also suggested that using CE Ferulic serum led to a greater improvement in erythema index [20]. The accelerated healing process might be achieved through the blockage of laser treatment‐induced down regulation of basic fibroblast growth factor (bFGF) by vitamin C, E, and ferulic acid [14].

This trial showed that the side receiving the CE Ferulic serum had a greater reduction in MI compared with the NS‐treated side, which indicates antioxidant serum may improve hyperpigmentation stemmed from ablative CO_2_ laser treatment for atrophic acne scars. These findings are consistent with a previous randomized, split‐face trial among Korean patients with environment‐induced skin pigmentation, where the postoperative use of a combination of vitamin C, vitamin E, and ferulic acid exhibited a significantly greater reduction in MI compared with placebo following a non‐ablative laser procedure [15]. We speculate that the reparative effects of CE Ferulic serum may lie in its reduction of oxidative stress response after laser treatment, thereby decreasing postoperative inflammation and exudation.

In this trial, a better capability of retaining skin water and preventing water loss was observed on the CE Ferulic‐treated side compared to the NS side, especially when applied twice daily. Ablative CO_2_ laser treatment damages the stratum corneum and epidermis, leading to a decrease in skin hydration level and an increase in TEWL [2]. The results of this trial suggest that the CE Ferulic serum was beneficial in maintaining skin hydration following laser treatment. This study also showed the antioxidant serum had an overall favorable safety and tolerability profile, which was similarly reported in a retrospective study in the US [16].

A limitation of this study was that the patients applied the treatment by themselves, which could have led to variations in application technique and consistency. However, this self‐application approach closely mirrors real‐world scenarios, allowing for a more practical evaluation of the effectiveness of the treatment. Additionally, due to the relatively small sample size, we did not identify statistically significant differences of improvement between two subgroups. Lastly, this study primarily focused on objective outcomes and did not include subjective measures such as patient satisfaction. Future research is warranted to explore patient experience more comprehensively.

The findings of the present study highlight the potential of CE Ferulic serum as an effective addition to post‐laser treatment skincare regimens. By incorporating this professional‐grade product into their routine, patients can benefit from reduced side effects of ablative CO_2_ laser treatment, shortened wound healing, and optimal hydration. This is particularly meaningful for patients with acne scars, as existing evidence has already associated these benefits with better scarring treatment outcomes [21, 22, 23]. This evidence‐based Integrated SkinCare strategy has been supported by numerous trials and studies, with a wide range of combinations of professional skincare products and aesthetic procedures [14, 15, 24, 25]. Future research should strive to expand the potential applications of CE Ferulic serum in the field of skincare and explore whether other Integrated SkinCare strategy combinations are appropriate for the Chinese population.

## 5 Conclusion

In conclusion, this randomized, investigator‐blinded, split‐face, controlled trial found that a combination of vitamin C, E, and ferulic acid helped to improve wound healing, reduce erythema and melanin levels, and maintain skin barrier function following ablative fractional CO_2_ laser treatment compared with normal saline, suggesting its potential to promote skin repair and prevent post‐inflammatory hyperpigmentation.

## Author Contributions

All authors contributed significantly to the work. Yu Shi and Sijia Xu were responsible for data curation and contributed equally to the writing of the original draft. Wei Zhang led the project administration, as well as reviewing and editing the manuscript. All authors reviewed and approved the final manuscript.

## Funding

This work was supported by L'Oreal.

## Ethics Statement

This study has been approved by the Ethics Committee of Shanghai Skin Disease Hospital in February 2023 (no. 2023‐02 (scientific)).

## Consent

Written informed consent and photo consent were obtained from each subject.

## Conflicts of Interest

The authors declare the following potential conflicts of interest: The trial associated with this manuscript was funded by L'Oréal China. Study materials, including CE Ferulic serum, were provided by SkinCeuticals Inc. These entities had no role in the design, execution, analysis, or interpretation of the study results.

## Supporting information


**Table S1:** Stage of scabbing on Days 3, 7, 14 overall and by subgroup.

## Data Availability

The data that support the findings of this study are available from the corresponding author upon reasonable request.
