# Supplementary material for: Reparative Effects of a Topical Antioxidant Serum Containing Vitamin C, Vitamin E, and Ferulic Acid After Ablative Fractional CO 2 Laser Treatment for Atrophic Acne Scars: A Randomized, Investigator‐Blinded, Split‐Face, Controlled Trial
Source: J Cosmet Dermatol. 2026 Jan 11;25(1):e70634. doi: 10.1111/jocd.70634 (PMC12793811; doi:10.1111/jocd.70634)
Supplement: Supplementary file 1 — Table S1: Stage of scabbing on Days 3, 7, 14 overall and by subgroup. [file JOCD-25-e70634-s001.docx]

Table S1. Stage of scabbing on day 3, 7, 14 overall and by subgroup

| **Stage of scabbing** | **Overall**  **(N=64)** | | **Once-daily subgroup (N=31)** | | **Twice-daily subgroup (N=33)** | |
| --- | --- | --- | --- | --- | --- | --- |
|  | CE Ferulic | NS | CE Ferulic | NS | CE Ferulic | NS |
| **Day 3** | | | | | | |
| Scab formation, n(%) | 63 (98.44%) | 62 (96.88%) | 31 (100%) | 31 (100%) | 32 (96.97%) | 31 (93.94%) |
| Partial scab detachment, n(%) | 1 (1.56%) | 2 (3.12%) | 0 (0%) | 0 (0%) | 1 (3.03%) | 2 (6.06%) |
| Complete scab detachment, n(%) | 0 (0%) | 0 (0%) | 0 (0%) | 0 (0%) | 0 (0%) | 0 (0%) |
| **Day 7** | | | | | | |
| Scab formation, n(%) | 0 (0%) | 1 (1.56%) | 0 (0%) | 1 (3.2%) | 0 (0%) | 0 (0%) |
| Partial scab detachment, n(%) | 25 (39.06%) | 41 (64.06%) | 12 (38.7%) | 21 (67.7%) | 13 (39.4%) | 20 (60.6%) |
| Complete scab detachment, n(%) | 39 (60.94%) | 22 (34.38%) | 19 (61.3%) | 9 (29.0%) | 20 (60.6%) | 13 (39.4%) |
| **Day 14** | | | | | | |
| Scab formation, n(%) | 0 (0%) | 0 (0%) | 0 (0%) | 0 (0%) | 0 (0%) | 0 (0%) |
| Partial scab detachment, n(%) | 0 (0%) | 2 (3.12%) | 0 (0%) | 1 (3.23%) | 0 (0%) | 1 (3.03%) |
| Complete scab detachment, n(%) | 64 (100%) | 62 (96.88%) | 31 (100%) | 30 (96.77%) | 33 (100%) | 32 (96.97%) |
